# The changing landscape of pharmaceutical alternatives to the unregulated drug supply during COVID-19

**DOI:** 10.1186/s12954-022-00657-x

**Published:** 2022-07-14

**Authors:** Karen McCrae, Stephanie Glegg, Marie-Éve Goyer, Bernard Le Foll, Rupi Brar, Christy Sutherland, Nadia Fairbairn

**Affiliations:** 1 British Columbia Centre On Substance Use, 400-1045 Howe Street, Vancouver, BC V6Z 2A9 Canada; 2 Department of Medicine, University of British Columbia, St. Paul’s Hospital, 608-1081 Burrard Street, Vancouver, BC V6Z 1Y6 Canada; 3 PHS Community Services, 9 E Hastings St, Vancouver, BC V6A 1M9 Canada; 4 Department of Family Medicine, University of British Columbia, 5950 University Blvd, Vancouver, BC V6T 2A1 Canada; 5 Vancouver Coastal Health Authority, 801-601 West Broadway, Vancouver, BC V5Z 4C2 Canada; 6 Family Medicine and Emergency Department, University of Montréal, Montréal, QC H3T 1J4 Canada; 7 Translational Addiction Research Laboratory, Centre for Addiction and Mental Health, 33 Ursula Franklin Street, Toronto, ON M5S2S1 Canada; 8 Campbell Family Mental Health Research Institute Centre for Addiction and Mental Health, Ursula Franklin Street, Toronto, ON M5S2S1 Canada; 9 Department of Psychiatry, University of Toronto, 250 College Street, Toronto, ON M5T1R8 Canada; 10 Faculty of Medicine, Institute of Medical Science, University of Toronto, Medical Sciences Building, 1 King’s College Circle, Room 2374, Toronto, ON M5S 1A8 Canada; 11 Department of Family and Community Medicine, University of Toronto, 500 University Avenue, 5th Floor, Toronto, ON M5G 1V7 Canada; 12 Department of Pharmacology and Toxicology, University of Toronto, 27 King’s College Circle, Toronto, ON M5S3H7 Canada

**Keywords:** Safe supply, Substance use, Harm reduction, Injectable opioid agonist treatment, COVID-19 pandemic, Canada

## Abstract

**Background:**

The dual COVID-19 and overdose emergencies amplified strain on healthcare systems tasked with responding to both. One downstream consequence of the pandemic in the USA and Canada was a surge in drug overdoses resulting from public health-restricted access to services and an increasingly toxic unregulated drug supply. This study aimed to describe changes implemented by programs prescribing pharmaceutical alternatives to the drug supply during the early stages of the COVID-19 pandemic.

**Methods:**

An environmental scan used surveys and qualitative interviews with service providers across Canada to examine pharmaceutical alternative prescribing practices and programs before and during the pandemic. This study summarized the nature, frequency, and reasons for pandemic-driven service delivery changes using directed content analysis, counts, and thematic analysis.

**Results:**

Eighty-two of the 103 participating sites reported 1193 unique changes in physical space (368), client protocols (347), program operations (342), ancillary services (127), and staffing (90). Four qualitative themes describing the reasons for these changes emerged, namely (1) decreasing risk of COVID-19 infection; (2) decreasing risk of overdose; (3) prioritizing acute care of COVID-19 patients; and (4) improving client access to treatment.

**Conclusions:**

While most changes were aimed at decreasing risk of COVID-19 infection, some were found to be at odds with the measures needed to combat the overdose crisis; others met dual objectives of decreased risk of both overdose and infection. Further research should examine which changes should be kept or reversed once COVID-19-related public health measures are lifted.

## Background

People who use drugs (PWUD) have experienced record high rates of fatal overdoses and other harms during the COVID-19 pandemic: In the USA, over 94,000 drug overdose deaths were reported in the 12 months ending in January 2021, the highest number of overdose deaths ever recorded in a 12-month period—far eclipsing the 72,000 individuals who died in previous 12-month period [1]. Similarly in Canada, 1,705 individuals died of apparent opioid overdose between July and September 2020, the highest quarterly count since national surveillance began in 2016 [2].

In order to respond to the spike in overdoses that corresponded with the start of the COVID-19 pandemic and address pandemic-related public health restrictions, addiction healthcare practitioners and service providers adapted their programming in a very short period of time—sometimes in drastic ways. For example, physical distancing requirements resulted in a rapid shift to telehealth, staff, and space were sometimes redistributed to prioritize treatment of COVID-19, and in Canada, pharmaceutical-grade alternatives to the unregulated drug supply (i.e., “safer supply”) started becoming more widely available [3]. Although frontline services and treatment providers navigated the oftentimes conflicting needs presented by the opioid overdose crisis and the pandemic with little direction from public health officials, guidance on developing safer supply programs began to emerge and expand in several jurisdictions [4–7].

During the early days of the pandemic, researchers at the British Columbia Centre on Substance Use (BCCSU) on behalf of the Canadian Research Initiative in Substance Misuse (CRISM) were preparing to conduct a third annual national environmental scan to monitor the emergence of iOAT (i.e., injectable hydromorphone- or diacetylmorphine-based opioid agonist treatment, offered in structured and typically resource-intensive settings with stringent oversight [8]). This scan also included tablet-based iOAT (TiOAT) programs, which are provided in a supervised low-threshold setting such as a supervised consumption site with a flexible dosing model (i.e., doses are not individualized for the patient and no induction/titration periods are provided) [9]. Given the rapidly changing landscape and lack of available data to guide public health decision-making in the context of dual public health emergencies, the scope of the national environmental scan was expanded to document changes that participating sites made between March 1 and May 1, 2020, and to include sites newly engaged in safer supply prescribing. In Canada, iOAT, TiOAT, and safer supply are currently provided at: (1) comprehensive and dedicated programs in stand-alone facilities or acute care centers, (2) facilities embedded within existing community health clinics, harm reduction programs, and housing programs (note that safer supply is offered in a more diverse range of settings than iOAT and TiOAT, including youth and mental health services and perinatal care, among others [10]), (3) pharmacy-based sites, whereby services are provided in existing clinics with supervision of administration provided by trained pharmacists at select pharmacy locations, and (4) select hospitals, whereby treatment is initiated in hospital with transfer to community-based prescribers at discharge [11, 12]. Safer supply may also be accessed via healthcare practitioners who prescribe it as part of their regular medical practice [12].

The purpose of the current subset of the environmental scan’s analysis was to identify the service delivery changes implemented by sites offering pharmaceutical alternatives (i.e., iOAT, TiOAT, safer supply) in Canada, early on in the COVID-19 pandemic. While the dramatic 285% increase in the number of sites offering safer supply between March 1 and May 1, 2020 is described in detail elsewhere [10], this article is focused on adjustments to staffing, ancillary services, program operations, use of physical space, and client protocols. This analysis will be of particular interest to policy makers and public health officials, prescribers, and other healthcare providers to inform the nature and impact of COVID-19 responses on addiction care and harm reduction service implementation.

## Methods

The annual, national, mixed methods environmental scan used a written survey and qualitative interviews with service providers to examine iOAT, TiOAT, and safer supply prescribing practices and services across Canada. This study was approved by the University of British Columbia/Providence Health Care Joint Research Ethics Board, under the purview of program evaluation.

The majority of participants were recruited via email or social media outreach by identifying grant recipients, authors, and clinical and research project members doing work on iOAT, TiOAT, and/or safer supply through scoping Web, primary, and gray literature searches. Search terms included injectable opioid agonist treatment; tablet injectable opioid agonist treatment; iOAT; TiOAT; and safe(r) supply. No search limits were applied. We screened Google search results using the title and summary text of entries and checked which programs were operational during our scan dates. Searches were repeated periodically through the fall of 2020. Outreach also included provincial and national professional associations, research networks, clinical guideline committees, peer and advocacy groups, health and service providers, federal and provincial governments, and health authorities. A small number of participants [3] were identified through snowball sampling and word of mouth [13]. All identified programs were invited to participate, and surveys were only sent out to individuals who indicated interest in participating. We asked that each site nominates one participant (e.g., nurse, physician, clinic manager), although each participant could report on any number of sites at which they provided services through a single survey or interview. There were no target numbers of contacts across respondent categories.

The written survey was based on the previous CRISM national environmental scans of iOAT programs across Canada [8]. Open-text survey questions relevant to this sub-analysis asked about any pandemic-driven changes made to services. Individual telephone or Zoom interviews were used to clarify survey responses as needed and to gather supplementary qualitative data on the barriers, facilitators, and impacts of service implementation and operation. Prior to publication, participants were offered the opportunity to verify the accuracy of their answers via email or telephone, regardless of whether they submitted a survey, participated in an interview, or completed both. These flexible participation options were provided in order to reduce respondent burden in the context of the pandemic. The methods are described in more detail elsewhere [10].

The present sub-analysis involved: (1) transcribing audio-recorded interviews and importing them into NVivo [14]; (2) coding survey and interview data on reported changes using directed content analysis [15] based on five a priori categories, namely staffing, ancillary services, program operations, use of physical space, and client protocols; (3) producing frequency counts of individual changes within each a priori category; (4) using inductive thematic analysis [16] to identify themes on the reasons for the reported changes; and (5) validating these themes against the synthesized survey data about the changes and against the codes within each a priori category to confirm their trustworthiness [16]. Thematic analysis involved grouping into identifiable patterns the verbatim statements and repeating ideas from the data that described the factors underlying the reported changes, and interpreting them in the context of their reported chronology [17].

## Results

In total, 103 sites from BC, Alberta, Ontario, Quebec, and the Atlantic provinces were represented in this study, of which 82 (19 iOAT, 3 TiOAT, and 60 safer supply) provided data related to 1,280 unique changes made in response to COVID-19. These data are summarized in Table 1.

**Table 1:** Changes to pharmaceutical alternative services as a result of COVID-19

Changes to services	iOAT *N* = 19	TiOAT *N* = 3	SS *N* = 60	Total *N* = 82
Staffing totals	12	1	77	90
Added positions	8	1	29	
Reduced/redeployed positions	3	0	24	
Staff worked from home	1	0	23	
Staff hours staggered	0	0	1	
Ancillary services totals	32	4	91	133
Referrals delayed/canceled	5	3	10	
Community meetings eliminated	2	0	1	
Care moved outdoors	1	0	2	
Virtual ancillary care	12	1	45	
Reduced services/activities	5	0	21	
Bulletin board, books, fabric, art supplies removed	3	0	14	
Food services reduced/take-out only	4	0	4	
Program operations totals	102	18	222	342
No new client intakes	12	1	7	
Screening upon entry or pre-screened by phone	17	3	35	
Staff required to wear PPE	15	3	30	
Clients encouraged/required to wear PPE	16	3	31	
Increased access to/expectation of hand sanitizer use/handwashing upon entry	15	3	23	
Reduced laboratory hours	6	1	7	
Video/phone appointments	12	3	44	
Reduction in frequency patients seen by physician	6	1	32	
One clinician covers all in-person appointments	0	0	2	
Mobile team created to support clients at home	1	0	9	
Limited/restricted outreach to clients at home	2	0	2	
Use of physical space totals	121	21	226	368
Reduced/repositioned furniture	14	3	23	
Reduced # of clients in clinic/injection room	18	3	32	
Reduced # clients in chill out/waiting/shared activities room	14	3	23	
Bathrooms closed to patients	2	2	2	
Client entry denied/discouraged for non-treatment purposes	10	3	16	
Creation/use of isolation room	8	0	12	
Physical distancing enforced	13	3	20	
Plexiglass installed	6	0	16	
Additional/enhanced cleaning	18	3	52	
Decreased amount of space available for treatment	5	0	15	
Limited tours/fellowship placements	13	1	15	
Client protocols totals	129	13	205	347
Reduction in urine drug test frequency	12	1	26	
Swabbing clients suspected of being COVID+	11	1	16	
Stop/reduce post-dose adverse reaction monitoring period	15	0	13	
Take-home/carries to stable patients	13	1	27	
Reduced # of injections (with patient consent)	7	NA	NA	
Less frequent dose pickup	2	1	12	
Extending/renewing prescriptions at the pharmacy	0	0	14	
TiOAT/iOAT patients offered safe supply	6	1	NA	
Daily delivery	3	1	25	
Shorter titration schedule/expedited dose increases	8	0	33	
Transition to oral OAT encouraged	10	1	1	
Pre-filled syringes	2	NA	NA	
Provide/replace oral hydromorphone in addition to iOAT	14	NA	NA	
SROM administered as full capsule (no sprinkles)	12	3	16	
Changed overdose response/Code Blue procedures	14	3	22	

iOAT = injectable opioid agonist treatment; TiOAT = tablet-based iOAT; SS = safer supply; OAT = opioid agonist treatment; SROM = slow-release oral morphine; PPE = personal protective equipment; NA = not applicable

A total of 50 individual participants provided data. Two participants completed the written survey only (representing 3 sites), 17 provided an interview only (39 sites), and 31 completed both (60 sites), including physicians, medical residents, nurse practitioners, nurses, medical directors, managers, coordinators, leads, and knowledge translation and evaluation officers. Survey respondents consulted an additional 116 colleagues; along with many of the roles listed above were medical students, nursing assistants, pharmacists, pharmacy technicians, directors, community action team members, and mental health, social, outreach, community liaison, personal support, and peer workers.

Four qualitative themes emerged from the interview data to describe the reasons for these changes: (1) decreasing risk of COVID-19 infection; (2) reducing overdose risk; (3) prioritizing acute care of COVID-19 patients; and (4) improving client access to treatment.

### Decreasing risk of COVID-19 infection

Changes to services that fell within this theme were typically initiated first, and designed to support self-isolation of clients who tested positive or were awaiting results, physical distancing, screening for COVID-19, and limiting SARS-CoV2 transmission during necessary close contact between staff and clients/patients. Many of these changes were mandated by public health officials, health authorities, and/or the programs’ parent organizations. In addition to common infection control practices, such as increased handwashing, and providing personal protective equipment (PPE), changes were also made to the environment to help prevent transmission (e.g., erecting plexiglass barriers and designating isolation spaces).

In some cases, these modifications interrupted the culture of the clinic and the sense of community that had been established. As one respondent at a safer supply program noted:

*“It’s harder to build relationships with folks when you're not able to see them in person as much. We used to have food out all the time, we had a book nook, we had kids’ toys, you know, there was stuff here, stuff to make it feel comfortable and cozy and welcoming, and it’s harder when we’re not seeing people in person as much.” – Participant 29*

### Decreasing risk of overdose

Pursuing strategies to reduce overdoses became a key driver of change at some sites after policies were implemented to decrease risk of COVID-19 transmission. This shift was reported to be driven by the increasing mortality and morbidity associated with the use of unregulated drugs in the early days of the pandemic. Perhaps, the most notable change in this category is reflected by the number of sites that provided safer supply across Canada, which expanded by 60 between March 1 and May 1, 2020 (21 to 81). Participants reported recognizing the heightened risks their clients faced from the increasingly toxic drug supply and the lack of supports available to PWUD as a consequence of public health restrictions [10]. Strategies such as increasing medication dosages and expanding the range of available medications became paramount for addressing overdose risk after urgent COVID-19-targeted actions to reduce risk were implemented. Some sites reported transitioning iOAT clients to oral treatment by way of safer supply, while others replaced contaminated street drugs with prescribed alternatives simply to prevent death. As one respondent noted:

*“The biggest thing is people aren’t dying [of overdose]. It sounds quite frank, but it’s just the truth, right. People aren’t dying. People are able to access a supply of drugs that meets their needs and their goals, and they’re not having to worry about, you know, ‘Is this the dose that’s going to kill me?’ So that is, for me, and for many of them, the greatest thing ever – that they don’t have to worry day by day about that.” – Participant 2*

Another participant also noted the impact of safer supply on overdose rates in stark terms. When asked about the importance of safer supply for clients, they expressed:

*“[Safer supply is] outrageously important. Like it’s everything…I think its life and death. I think it’s also how we show respect and support. I think it’s a gateway not to drugs, but it’s a gateway to care and to human love and compassion and I think it is the barrier that causes a lot of people to become despaired and isolate from healthcare.” – Participant 36*

### Prioritizing acute care of COVID-19 patients

Strategies within this theme followed or were implemented concurrently with changes to address heightened overdose risk, as COVID-19 case counts rose. These approaches involved moving resources from existing services toward managing COVID-19 care, particularly at sites associated with hospitals or healthcare services. For example, staff were redeployed to COVID-19-related care teams or screening; services were relocated or merged to either create treatment space for COVID-19 patients, or to help ration the limited supply of personal protective equipment (PPE) available at the onset of the pandemic. One respondent at a safer supply program described the impact:

*“Because of COVID and challenges in supplying all three of those clinics with enough PPE, all of our staff was transitioned into our one main downtown site…What that practically means? I just have a lot less space and time in the clinic.” – Participant 3*

### Improving client access to treatment

Many changes that were meant to limit the transmission of COVID-19, such as decreasing the required frequency of in-person clinic visits, had cascading effects that decreased access to care. In response, service providers took action to ensure clients still had some access to services by making a series of changes. As one respondent expressed:

*“The goal, initially, was just to get them out of the clinic, decrease their frequency in the clinic. And then as the COVID response increased, we wanted to, with their agreement of course, keep them out of the clinic altogether just so they didn’t have to take transit, come into the building, etc. And so, the prescribing has changed.” – Participant 4*

At some sites, these changes centered around dosing. For example, some clients were transitioned from in-clinic injectable administration to oral administration (sometimes with options for take-home dosing), and two sites began providing vials of injectable hydromorphone for clients to draw up in syringes at home. At other sites, adaptations were made to communication processes (e.g., transitioning to virtual care, connecting more frequently with partnering stakeholders to reach or monitor clients) or to the location of care (e.g., establishing outreach programs), to meet clients in their communities more effectively. These strategies were seen by several respondents as adding tremendous value in understanding and connecting with clients, as described by one individual involved in a newly formed mobile outreach team:

“*For me, and I think for many that worked within that mobile unit, our ability to connect with clients changed dramatically after being welcomed into [the client’s] home, because, I mean, it’s another level of connection that you just can never get if you don’t see it.” – Participant 4*

## Discussion

This study found evidence of rapid transformation in service implementation and delivery early on in the COVID-19 pandemic. This transformation included a massive increase in the number of clinics (60) that started prescribing pharmaceutical alternatives to address the unintended negative consequences of the pandemic response, in addition to other rapid and significant changes to service provision across Canada. Changes were made particularly in relation to the way that physical space was managed, client protocols were implemented, and programs were operated.

However, measures to decrease the risk of COVID-19 were sometimes at odds with goals aimed at combating the opioid overdose crisis and providing adequate treatment for opioid use disorder. For example, some sites delayed or canceled ancillary services or referrals, eliminated community meetings, or moved to telehealth. These changes likely had some negative effects on clients, who may have felt less comfortable or able to access services as a result. Some particularly vulnerable individuals without access to the Internet or phones may have not been able to access services at all. As one participant noted,

*“It’s hard to keep connected to people when they’re moving around a lot and are less connected to us as a clinic, you know. Like their experience with us is more short-term so far, and a lot of these folks have only known us in this context of, you know, most appointments being done by phone or video call. So that makes me a bit sad, because you know, when our clinic was running before COVID, we always had food on-site, we had groups, it was a real environment of support and care.” – Participant 29*

Staffing changes that ensued from decreased capacity (whether from work-from-home policies or redeployment for COVID-19-related health care) and shifts in program operations likely also yielded less individualized care and attention for people on treatment. Some measures, such as staff and clients wearing PPE, physical distancing, and increased access to handwashing/sanitizing, almost certainly helped to reduce the risk of contracting SARS-CoV-2, likely with minimal harm to clients. However, other changes, such as stopping new client intakes, presented a major concern during the dual COVID-19 and opioid overdose crises as they likely had negative effects on the latter.

Interestingly, even in some clinics that went back to full or near-full capacity in 2020, some clients were hesitant to attend in person out of fear of contracting SARS-CoV-2—limiting their ability to access services even when those services were available to them. This finding highlights how some individuals’ desire to access in-person services changed during the pandemic, although more research will be required to determine the longevity of this change in preferences.

The largest number of reported changes is related to the way physical spaces were managed. These changes were typically made to reduce the number of people in clinics, for example through staggered access to treatment and other shared spaces, and by closing bathrooms to patients. The latter exacerbated an already serious lack of washroom access for people experiencing housing instability, particularly as many other businesses closed their doors during the pandemic [18]. While ~ 20% of sites reported the creation and/or use of an isolation room, not all sites had this option; a number of sites reported decreased treatment space because of reallocations in response to COVID-19. The loss of treatment space, at a time when physical distancing was necessary and mandated by public health bodies, was reported to further limit client access to services.

Qualitative data reinforced the negative impacts of these physical space-related changes on clients’ experiences and outcomes, as described by one respondent: *“I don’t know if [the changes to reduce COVID-19 transmission] have [supported people]. I mean, it’s what we need to do to try and keep everybody healthy and infection free. I’m not sure that they’ve supported people beyond that.” – Participant 3.*

However, the pandemic also led to new options for PWUD in terms of substance use service access and delivery. In addition to the 60 newly identified sites offering safer supply between March and May 2020, client protocols changed to include options that reduced burdens on clients. For example, a number of sites allowed whole capsule slow-release oral morphine to be administered without sprinkling in order to reduce patient/pharmacist interaction time. (Sprinkling is often required by regulatory colleges to reduce diversion [19].) New carry/take-home options, expedited titration schedules, and daily deliveries also reduced the amount of time clients and patients were required to spend at clinics, and enabled them to self-isolate and physically distance more effectively. While more research into the impact of these protocol changes is required, qualitative data highlighted some of their benefits, as illustrated by one respondent describing the impact of reducing the post-dose monitoring time: *“They hated the 15-min wait before, and now that it’s down to 5 min because of COVID, they like that way better. Waiting is a big thing; they hate the waiting.”– Participant 15.* However, it is important to note that not all of the changes to client protocols were seen as wholly beneficial. For instance, while expedited titration schedules were reported at ~ 50% of sites to assist some clients, one participant noted with unease that:

*“[For] some of my clients, I moved a little faster than I wanted to out of daily dosing because I didn’t want them, at the height of COVID, to have to be going into pharmacies on a daily basis.” – Participant 12*

This example highlights the conflicts faced by service and healthcare providers as they navigated responses to the dual health crises.

Interventions that were developed specifically to reduce overdose risk and/or reduce risk both of COVID-19 transmission and overdose (e.g., offering medications as take-home rather than witnessed dosing, offering virtual appointments, expanded access to medications through safer supply prescribing) should be continued post-pandemic to allow for evaluation of effectiveness for reducing substance-related harms. Most notably, we observed a rapid increase in the number of safer supply sites, a key pandemic-driven change that came about in an attempt to decrease risk of overdose and COVID-19 transmission. Preliminary research supports their purported benefits [20], and these programs should be continued and expanded to support ongoing and extensive evaluation of their impacts. Changes brought about solely to reduce COVID-19 transmission and prioritize acute care of COVID-19 patients (e.g., infection prevention and control practices, re-allocation of health services toward COVID-19 care) are less likely to benefit and, in some cases, may be detrimental, to clients in terms of substance use-related harms. These practices should be curtailed as COVID-19 transmission risk abates.

Several limitations to this study exist. Firstly, while we are confident that all providers of iOAT and TiOAT participated given the strong clinical provider networks that exist nationwide, it is uncertain what proportion of safer supply providers was reached as any medical care provider can prescribe safer supply as part of their regular medical practice [12]. Furthermore, we are aware of safer supply providers who did not participate due to workload demands and/or fear of consequences from colleagues, regulatory bodies, or regional governments. The ambiguous definition of safer supply within the prescriber community may have also let to some uncertainty on whether prescribing practices would qualify, and several sites that began prescribing after the reference date (May 1, 2020) were excluded. Not all participants completed both a survey and interview because of time constraints related to their clinical and operational responsibilities, which intensified during the pandemic. The survey asked specifically about changes to programs in response to COVID-19, while the interviews brought to light the reasons for them. This limitation likely resulted in an undercount of the changes and limited the gathering of additional data from survey-only participants that could explain the drivers of the changes more thoroughly. Lastly, the impacts of these changes on client care and satisfaction were not probed directly, given the early stage of the pandemic, although some of these impacts were articulated spontaneously by participants. Further research should examine this topic carefully, to inform policy makers and healthcare providers about which changes should be kept or abandoned in order to ensure the best possible client care.

## Conclusions

This study found that clinics providing pharmaceutical alternatives to the street drug supply in Canada were able to adapt and transform service implementation and delivery early on COVID-19 pandemic. While most changes were aimed at decreasing risk of COVID-19 infection, some were found to be at odds with the measures needed to combat the overdose crisis. Others met dual objectives, aiming to decrease risk of both overdose and infection. While further research is needed to examine which changes should be kept or reversed in a post-COVID environment, these findings highlight the potential for rapid change in clinics focused on treating addiction and responding to the overdose crisis and are highly relevant in light of the current public health situation.

## Data Availability

Reasonable requests for the anonymized data may be directed to the authors.
